# Expression in grasses of multiple transgenes for degradation of munitions compounds on live‐fire training ranges

**DOI:** 10.1111/pbi.12661

**Published:** 2016-12-29

**Authors:** Long Zhang, Ryan Routsong, Quyen Nguyen, Elizabeth L. Rylott, Neil C. Bruce, Stuart E. Strand

**Affiliations:** ^1^ Department of Civil and Environmental Engineering University of Washington Seattle WA USA; ^2^ CNAP Department of Biology University of York York UK

**Keywords:** phytoremediation, RDX, TNT, switchgrass, monocot promoters, stacked genes

## Abstract

The deposition of toxic munitions compounds, such as hexahydro‐1, 3, 5‐trinitro‐1, 3, 5‐triazine (RDX), on soils around targets in live‐fire training ranges is an important source of groundwater contamination. Plants take up RDX but do not significantly degrade it. Reported here is the transformation of two perennial grass species, switchgrass (*Panicum virgatum*) and creeping bentgrass (*Agrostis stolonifera*), with the genes for degradation of RDX. These species possess a number of agronomic traits making them well equipped for the uptake and removal of RDX from root zone leachates. Transformation vectors were constructed with *xplA* and *xplB*, which confer the ability to degrade RDX, and *nfsI*, which encodes a nitroreductase for the detoxification of the co‐contaminating explosive 2, 4, 6‐trinitrotoluene (TNT). The vectors were transformed into the grass species using *Agrobacterium tumefaciens* infection. All transformed grass lines showing high transgene expression levels removed significantly more RDX from hydroponic solutions and retained significantly less RDX in their leaf tissues than wild‐type plants. Soil columns planted with the best‐performing switchgrass line were able to prevent leaching of RDX through a 0.5‐m root zone. These plants represent a promising plant biotechnology to sustainably remove RDX from training range soil, thus preventing contamination of groundwater.

## Introduction

Continual military activity over nearly a century has resulted in the contamination of land and groundwater by high explosives, in particular, hexahydro‐1, 3, 5‐trinitro‐1, 3, 5‐triazine (RDX) and 2, 4, 6‐trinitrotoluene (TNT). These compounds enter the environment through manufacturing, military use and the decommissioning of outdated explosives. Human toxicity associated with TNT includes aplastic anaemia, and hepatitis, while RDX affects the central nervous system (Deng *et al*., 2014). Both RDX and TNT are listed by the EPA as possible human carcinogens. More than 100 military bases and explosive‐manufacturing facilities in the USA are now contaminated with these chemicals, which are highly recalcitrant to degradation in the environment. Live‐fire training at military bases has resulted in the contamination of soils around targets with particulates of RDX and TNT, which leach into the soil environment. The groundwater at these sites is at risk of contamination by the relatively mobile RDX, increasing the likelihood that the health risk will spread to drinking water sources beyond the military bases (Rivera *et al*., 1998). In contrast to RDX, TNT binds tightly to soil surfaces and is a lesser threat to groundwater. However, TNT is highly phytotoxic and its presence as a co‐contaminant can hinder clean‐up operations for RDX (Rylott and Bruce, 2009).

In the USA, the clean‐up of active ranges contaminated with explosives has been estimated by the US Department of Defense to cost between US$16 billion and US$165 billion (United States General Accounting Office 2004). A sustainable and potentially low‐cost alternative for remediating munitions contaminated soils is phytoremediation: the use of plants to degrade the pollutants. Although plants are able to take up RDX, translocating it to the aerial tissues (Brentner *et al*., 2010), degradation of RDX by plants is low (Just and Schnoor, 2004; Winfield *et al*., 2004), resulting in the persistence of RDX in soil environments. Studies in poplar tissues (*Populus deltoides* × *nigra* DN‐34) show that RDX in leaf tissue is partially reduced to hexahydro‐1‐nitroso‐3, 5‐dinitro‐1, 3, 5‐triazine (MNX) and hexahydro‐1, 3‐dinitroso‐5‐nitro‐1, 3, 5‐triazine (DNX). Further transformation of RDX, MNX and DNX results in the formation of formaldehyde, methanol and carbon dioxide through a photolytic mechanism (Van Aken *et al*., 2004).

The process of TNT uptake by plants, observed in a number of species, is hampered by its acute phytotoxicity (Johnston *et al*., 2015). Although plants have only a limited ability to detoxify TNT, the biochemical pathways involved have been well studied in Arabidopsis. Following uptake, TNT is transformed by oxophytodienoate reductases (OPRs; Beynon *et al*., 2009) and then conjugated by uridine diphosphate glycosyltransferases (UGTs; Gandia‐Herrero *et al*., 2008). Conjugation directly to the TNT molecule by glutathione transferases (GSTs) has also been demonstrated (Gunning *et al*., 2014). In order to increase the effectiveness of phytoremediation, it is possible to express transgenes involved in metabolism, uptake or transport of specific pollutants in genetically modified plants. This strategy combines the advantages of selected plant species such as high biomass and transpiration rates, and ease of cultivation; with the diverse catabolic capabilities of bacteria.

Bacterial genes encoding enzymes for the degradation and transformation of RDX and TNT, respectively, have been identified. *Rhodococcus rhodochrous* strain 11Y was isolated from explosive‐contaminated soils and found to grow on RDX as the sole source of nitrogen. The RDX degradation system was subsequently characterized and found to comprise a novel fused flavodoxin–cytochrome P450 XplA and partnering flavodoxin reductase XplB (Rylott *et al*., 2006, 2011b; Seth‐Smith *et al*., 2002). The *xplA* gene and its reductase partner *xplB* were expressed together in Arabidopsis (*Arabidopsis thaliana*) and the transformants found to remove RDX from liquid culture and soil leachate at rates significantly faster than those of untransformed plants (Jackson *et al*., 2007). The *nfsI* gene from *Enterobacter cloacae* encodes a nitroreductase (NR) that transforms TNT (Bryant and DeLuca, 1991). Arabidopsis plants engineered with *xplA, xplB* and *nfsI* were able to degrade RDX and detoxify TNT, suggesting that plants adapted to training range conditions could reduce RDX contamination *in situ* if the plants were similarly transformed (Rylott *et al*., 2011a).

Several perennial grass species are well adapted to the environmental conditions found on training range conditions in temperate regions (Palazzo *et al*., 2005). This study focuses on the expression of *xplA, xplB* and *nfsI* in switchgrass (*Panicum virgatum*) and creeping bentgrass (*Agrostis stolonifera*) for the phytoremediation of RDX and TNT in soils such as those found at training ranges in the USA. High‐throughput *Agrobacterium*‐mediated transformation techniques are well established for switchgrass (Li and Qu, 2011; Ramamoorthy and Kumar, 2012; Xi *et al*., 2009) and for creeping bentgrass (Zhou *et al*., 2013). Transformation vectors were constructed using a multiple gene transformation vector system, pNSAT, which was based on the pSAT versatile vector system (Chung *et al*., 2005). The pNSAT vectors all contained *xplA, xplB* and *nfsI* along with the selection marker gene, *hygromycin B phosphotransferase* (*hpt*), which were driven by either monocotyledon‐specific promoters or the 35s promoter. This is the first report of the genetic transformation of grasses for phytoremediation.

## Results

### Vector construction

**Figure 1:**
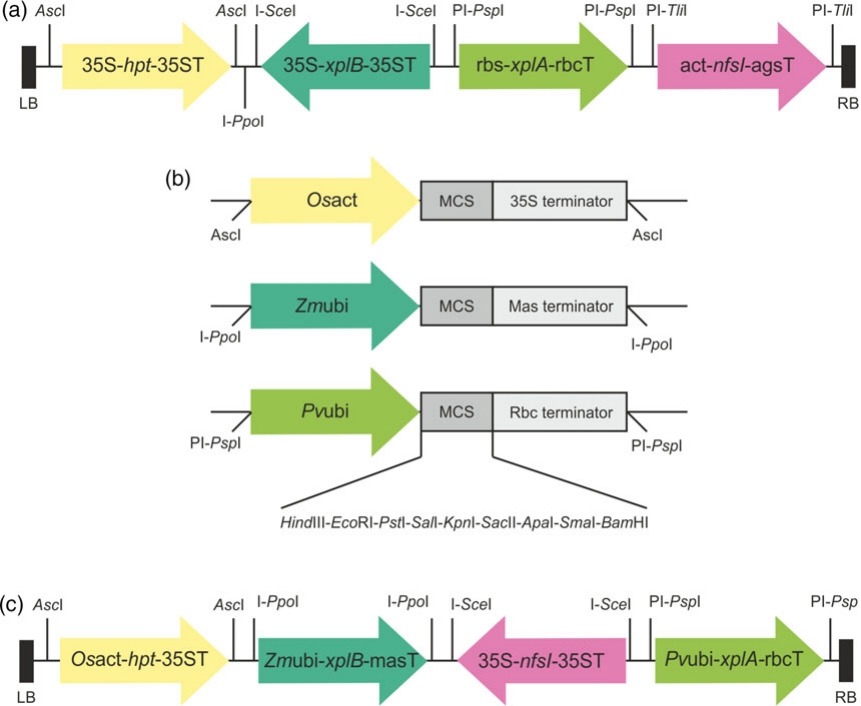
Construction of vectors for transformation of the grasses. (a) T‐DNA region of the binary vector plasmid pRCS2‐ABNR‐HR. The RDX degradation gene *xplA*, flavodoxin reductase gene *xplB* and TNT‐detoxifying nitroreductase gene *nfsI* were constructed into versatile cloning vector pSATs (Chung *et al*., 2005). Arrows show the direction of transcription. (b) The *Os*act, *Zm*ubi and *Pv*ubi promoters were used to replace the promoters in the pSAT vectors resulting in pNSAT1a, pNSAT3a and pNSAT6a, respectively. (c) T‐DNA region of the binary vector plasmid pRCS2‐NABNR. The *hpt*,* xplA, xplB* and *nfsI* genes were constructed into pNSAT1a, pNSAT6a, pNSAT3a and pSAT4a, respectively. The expression cassettes of these genes were integrated into the binary vector pPZP‐RCS2 to produce pRCS2‐NABNR. Abbreviations: 35s, CaMV 35s; rbc, Rubisco small subunit; act, actin; ags, agropine synthase; *Os*act, *Oryza sativa* actin promoter; *Zm*ubi, *Zea mays* ubiquitin promoter; *Pv*ubi, *Panicum virgatum* (switchgrass) ubiquitin promoter; RB left border; RB right border.

To produce grass lines expressing multiple transgenes, the pSAT vector series were selected (Chung *et al*., 2005) and further modified. In order to identify putative transformants, the *hpt* gene, which encodes resistance to hygromycin, was employed. Selection efficiency of transformants was further enhanced by replacing the promoter and terminator regions in the expression cassette ocs‐*hpt*‐ocs in pSAT1a with 35S promoter and terminator regions to produce the cassette pSAT1a‐35S‐*hpt*‐35T. The *xplA*,* xplB* and *nfsI* genes were inserted into pSAT6a, pSAT4a and pSAT7a to produce pSAT6a‐*xplA*, pSAT4a‐*xplB* and pSAT7a‐*nfsI*, respectively. Then, the expression cassettes of 35S‐*hpt*‐35ST, rbc‐*xplA*‐rbcT, 35S‐*xplB*‐35ST, act‐*nfsI*‐agsT were excised from the pSAT vectors with the appropriate homing endonucleases and sequentially inserted into the corresponding restriction sites of the binary vector pPZP‐RCS2 to produce pRCS2‐ABNR‐HR, as shown in Figure 1a.

The pSAT vector sets are tailored to function in dicot species, but to achieve optimal expression in monocot species, it is necessary to use native monocot promoters (Mann *et al*., 2012). The rice actin promoter (*Os*act) and maize ubiquitin promoter (*Zm*ubi) are widely used in monocot crops due to their ability to direct high levels of near constitutive gene expression (Cornejo *et al*., 1993; McElroy *et al*., 1990). The switchgrass ubiquitin promoter (*Pv*ubi) has strong constitutive expression in switchgrass and rice (Mann *et al*., 2011). These three promoters were used to replace the 35S promoter in pSAT1a‐35S, the mas promoter in pSAT3a and the rbc promoter in pSAT6a, producing pNSAT1a, pNSAT3a and pNSAT6a, respectively (Figure 1b). The selectable marker gene *hpt*, and the target genes *xplA*,* xplB* and *nfsI* were inserted into pNSAT1a, pNSAT6a, pNSAT3a and pNSAT4a to produce *Os*act‐*hpt*‐35ST, *Pv*ubi‐*xplA*‐rbcT, *Zm*ubi‐*xplB*‐masT and 35S‐*nfsI*‐35ST cassettes. These expression cassettes were then integrated into the corresponding homing restriction sites of pPZP‐RCS2 to produce pRCS2‐NABNR (Figure 1c).

### Functional evaluation of the pNSAT vectors

**Figure 2:**
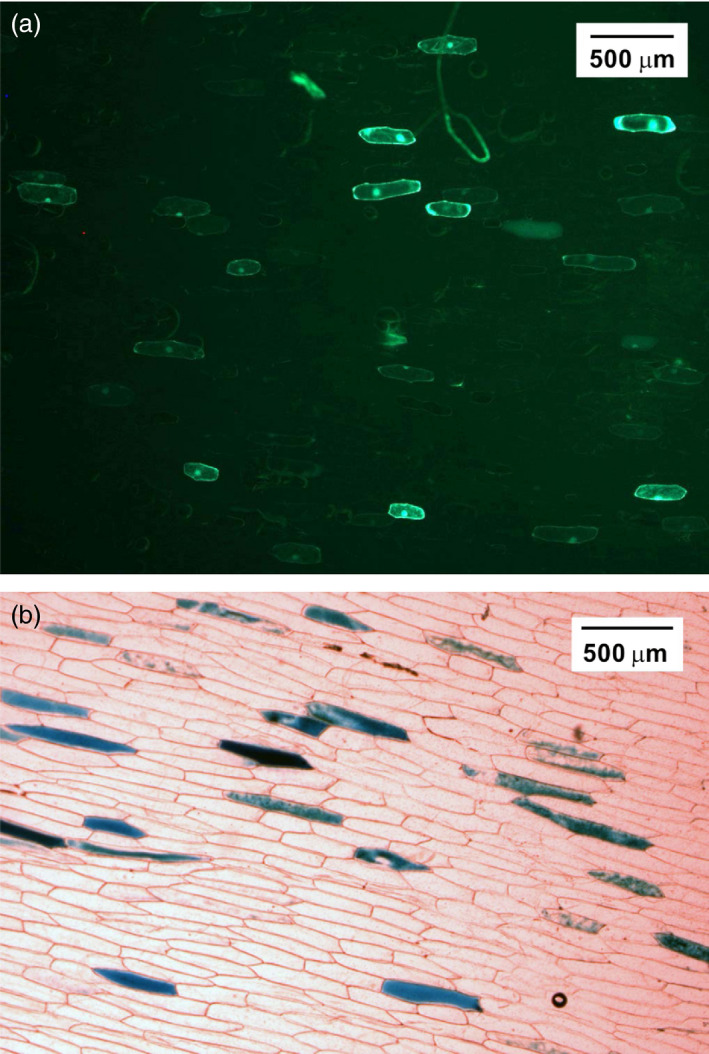
Functional evaluation of the pNSATs vectors using transient expression reporter genes in the cytosol of epidermal onion cells. (a) Fluorescence microscopy showing GPF expression following particle bombardment with pNSAT1a‐GFP (*Os*Act‐GPF‐35S). (b) Histochemical staining of GUS expression following particle bombardment with pNSAT3a/6a‐GUS (*Zm*Ubi‐GUS‐Mas; *Pv*Ubi‐GUS‐rbc).

To validate the functionality of the expression cassettes of pNSAT, the green fluorescent protein‐encoding *gfp* gene was inserted into pNSAT1a to produce pNSAT1a‐*gfp* and a second visual reporter gene (*gus*) encoding β‐glucuronidase was inserted into pNSAT3a and pNSAT6a to produce pNSAT3a‐*gus* and pNSAT6a‐*gus*. The GFP and GUS were transformed, separately in epidermal cells of onion using the biolistic method and transient expression of GFP and GUS was visualized using fluorescent and light microscopy, respectively. Detailed methods are provided in the Supplement. As shown in Figure 2, transient expression of GFP and GUS was observed in the cytosol of epidermal cells.

### Production of transgenic grasses and molecular analysis

**Figure 3:**
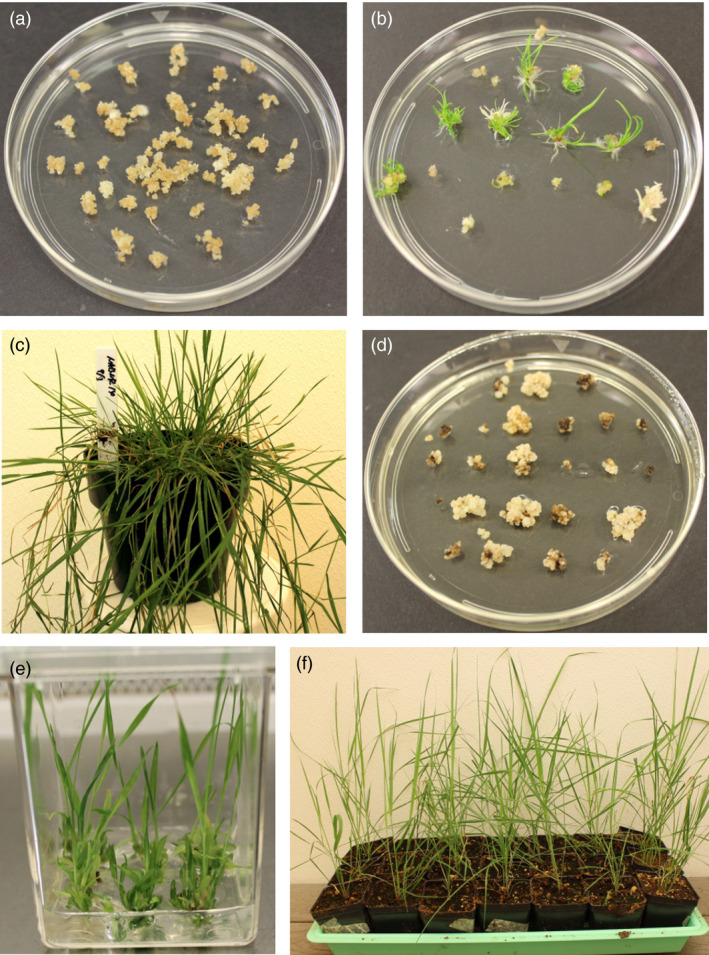
Production of transgenic creeping bentgrass and switchgrass. (a) Appearance of embryogenic calli of creeping bentgrass infected with Agrobacterium harbouring pRCS2‐NABNR after 3 weeks of culture on callus induction medium with hygromycin. (b) Hygromycin‐resistant calli on regeneration medium with hygromycin and (c) Transgenic plants in soil. (d) Appearance of embryogenic calli of switchgrass infected with Agrobacterium harbouring pRCS2‐NABNR after 4 weeks of culture on callus induction medium with hygromycin. (e) Hygromycin‐resistant plantlets on regeneration medium with hygromycin and f) Genetically transformed plants in soil.

Embryogenic calli of creeping bentgrass were infected with Agrobacterium strain EHA105 harbouring pRCS2‐ABNR‐HR or pRCS2‐NABNR and then placed on callus induction medium (CIM) containing the selection agent hygromycin (100 mg/L). After three weeks of selection, hygromycin‐resistant calli (Figure 3a) were transferred to regeneration medium containing hygromycin to develop plantlets, which were later transferred to soil (Figure 3b and c). To confirm the expression of the transgenes in the hygromycin‐resistant plantlets, qRT‐PCR analysis was conducted.

The results presented in Figure S1a show that all four pRCS2‐NABNR‐transformed creeping bentgrass lines expressed *xplA* to similar levels (*P* = 0.107), while the expression level of *xplB* in line N19 was more than twice that of the other lines (*P* = 0.018). The expression level of *nfsI* was much lower than that of *xplA* and *xplB*. Relative to *xplA* expression, the levels of *nfsI* transcript were 0.0233 ± 0.006, 0.0200 ± 0.0027, 0.0235 ± 0.0050 and 0.0110 ± 0.0019 for the lines N5, N14, N18 and N19, respectively. Western blot analysis of the transformed creeping bentgrass lines, shown in Figure S1b, revealed the presence of a single 60‐kDa band following immunoblot analysis using the XplA antibody, and corresponded in size to the XplA protein. A single 45‐kDa band was detected by immunoblot analysis using the XplB antibody, and corresponded in size to the XplB protein. Bands were not seen on blots probed using an antibody to the nitroreductase protein (NR), the product of *nfsI*.

**Figure 4:**
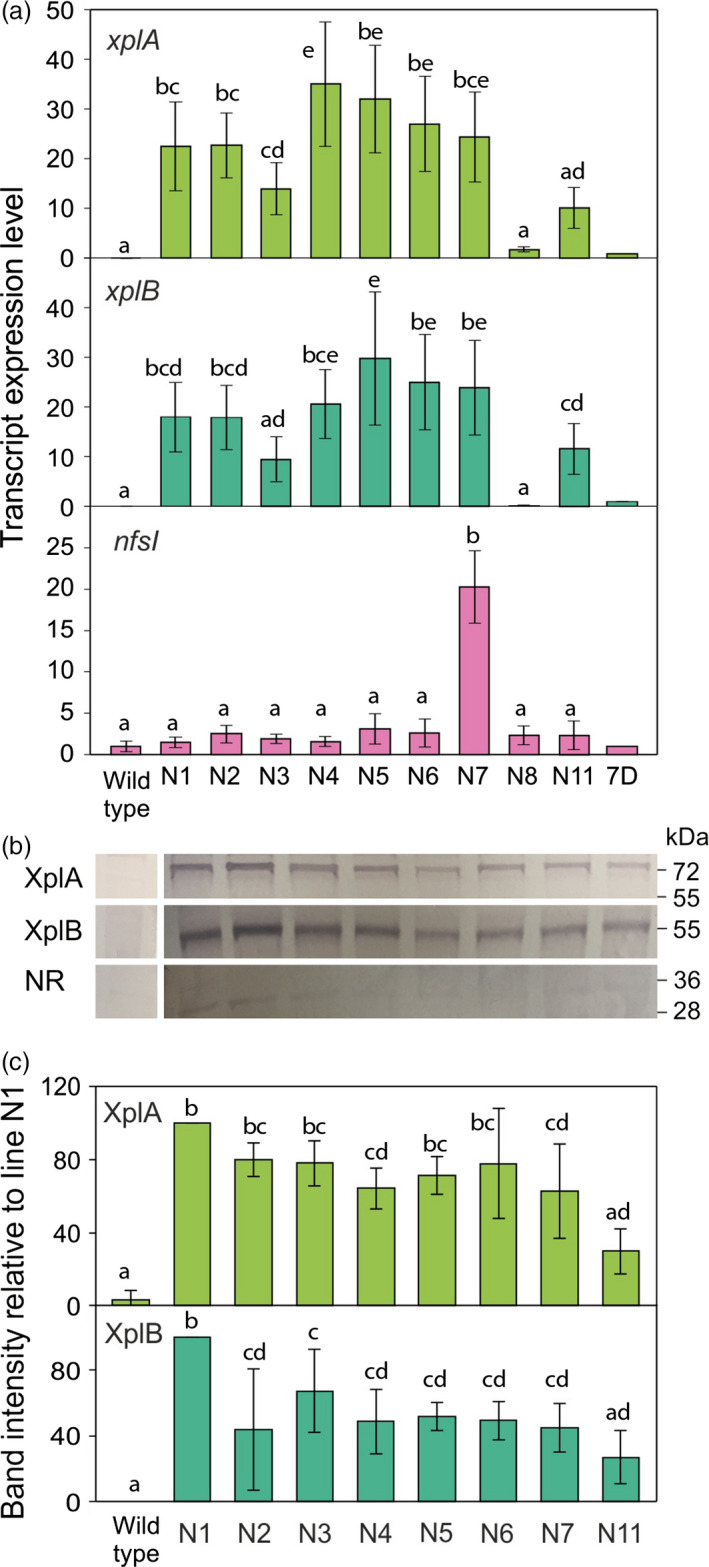
**Molecular characterization of** ***xplA*** **‐** ***xplB‐nfsI*** **‐transformed** switchgrass. (a) Transcript abundance measured using quantitative RT‐PCR on plant lines transformed with *xplA*,* xplB* and *nfsI*. Values were normalized to the switchgrass reference gene *eIF‐4a* (Gimeno *et al*., 2014). Arabidopsis values were normalized to the reference gene *ACT2*. All values are relative to the expression levels of the *xplA*‐*xplB*‐*nfsI* expressing *Arabidopsis* line 7D (Rylott *et al*., 2011a,b; *n* = 4 ± SE). (b) Western blot analysis on leaf blades of switchgrass lines expressing XplA, XplB and nitroreductase (NR) protein. (c) Band intensities were quantified for XplA and XplB expression. Levels were normalized to the Coomassie‐stained Rubisco large subunit; results are from three replicate blots ± SE.

To produce transformed switchgrass, friable type II embryogenic callus (Burris *et al*., 2009) was used for infection with Agrobacterium strain EHA105 harbouring the pRCS2‐NABNR vector. The calli were screened on CIM with 100 mg/L hygromycin for 2 weeks in the first round of selection. The surviving calli from the first selection round were transferred for further selection on CIM with 200 mg/L hygromycin (Figure 3d). Vigorously growing calli during the third round selection were transferred to regeneration medium containing 50 mg/L hygromycin for plant development (Figure 3e). After 2 months, healthy plants were transferred to soil (Figure 3f). To monitor transgene transcript levels, qRT‐PCR was conducted on the transformed switchgrass (Figure 4a). The relative levels of *xplA* and *xplB* were broadly similar across all transformed lines, with plant line N4 exhibiting the highest levels of expression for these transgenes.

The *xplA*‐*xplB*‐*nfsI* expressing Arabidopsis line 7D, published by Rylott *et al*. (2011a) was used as a guide; transgene expression in this line conferred significant ability to remove RDX and TNT from contaminated media (Rylott *et al*., 2011a); however, while the expression levels of *xplA* and *xplB* were all significantly higher than 7D in the transformed switchgrass lines, direct comparisons cannot be made. The levels of *nfsI* transcripts were significantly lower than for *xplA* and *xplB* in all the switchgrass lines, with the exception of line N7. In agreement with the relatively low transcript levels observed for *nfsI*, the western blot analyses presented in Figure 4b and c show that levels of NR were low when compared to the expression of XplA and XplB, and too low for band intensities to be accurately determined. As seen with the transcript levels for *xplA* and *xplB*, the protein levels of XplA and XplB were broadly similar across all the lines tested, with only a threefold difference in transcript and protein expression levels, with line N1 producing the highest levels of XplA and XplB protein.

### RDX uptake and degradation by transformed grasses

To determine the uptake rate of RDX by the transformed grasses, the plants were grown in liquid culture. The experiment used open test tubes to allow for maximum transpiration; physical losses of RDX were minimal due to the low volatility of RDX (Xiong *et al*., 2009).

**Figure 5:**
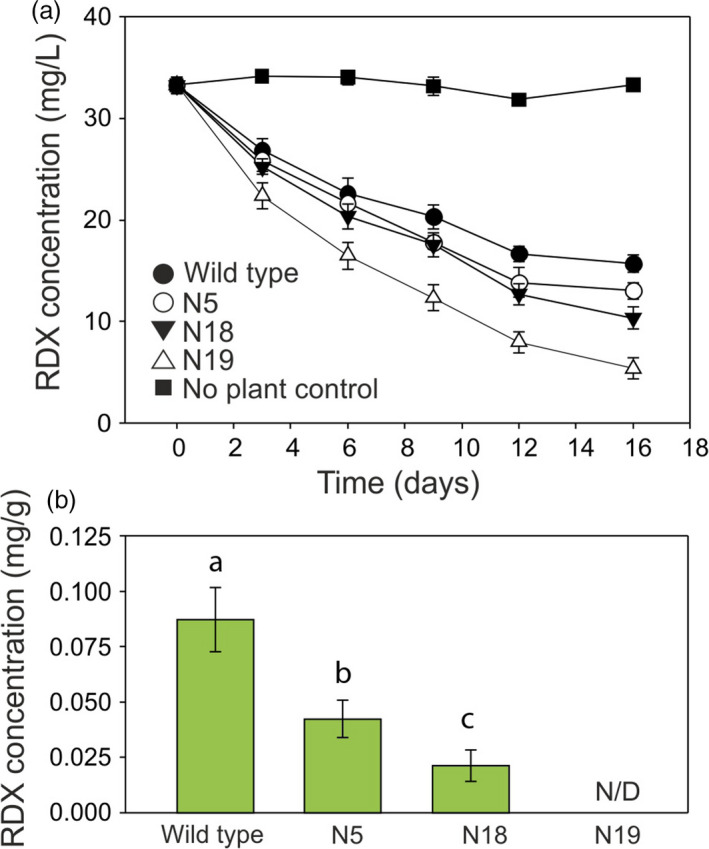
**Uptake of** **RDX** **by** ***xplA*** **‐** ***xplB‐nfsI*** **‐transformed creeping bentgrass grown in liquid culture**. (a) Concentration of RDX in culture medium over the course of the experiment. After three days, the medium from line N19 contained significantly (*P* = 0.010) less RDX than medium from wild‐type plants, and after nine days, lines N5 and N18 had also removed significantly more RDX from the media than wild type (*P* = 0.03 for N5 and 0.05 for N18). (b) Concentration of RDX in creeping bentgrass tissue after 16 days. Letters indicate that RDX concentrations in tissue were significantly different (*P* < 0.05) from other lines (*n* = 3 ± SE, N/D = none detected).

For creeping bentgrass, the RDX uptake from the medium is shown in Figure 5a. All three creeping bentgrass lines removed RDX from the medium faster than the wild‐type control line. After 3 days, the medium from creeping bentgrass line N19 contained significantly (*P* = 0.010) less RDX than medium from wild‐type plants and this difference increased during further culture. The RDX removal by line N19 was not only significantly greater than that of wild type, but also greater than lines N5 and N18 (*P* < 0.05). After 9 days of culture, lines N5 and 18 also removed significantly more RDX from the media than wild type (*P* = 0.03 for N5 and 0.05 for N18).

To determine accumulation of RDX in leaf tissue, RDX was extracted and analysed by HPLC at day 16 of the uptake experiments. RDX levels were highest in wild‐type creeping bentgrass tissues (Figure 5b), while lower levels of RDX were detected in plant lines N5 and N18; RDX was not detected in line N19.

The accumulation of RDX in the leaf tissue of wild‐type creeping bentgrass was correlated with a threefold depression of the growth of the plants compared with wild‐type plants grown in medium without RDX (Table 1). This decrease in biomass occurred despite the presence of sufficient nitrogen in the MS medium (20.6 mm ${\text{NH}}_{4}^{+}$ and 39.4 mm ${\text{NO}}_{3}^{-}$). In contrast, the transformed creeping bentgrass gained 1.5‐ to 2.5‐fold more biomass than wild‐type bentgrass when cultured in the presence of RDX.

**Table 1:** The effect of RDX on the growth of wild‐type and *xplA*‐*xplB*‐*nfsI*‐transformed creeping bentgrass

Plant line	Initial biomass (g)	Final biomass (g)	Biomass gain (g)
Control, no RDX	0.132 ± 0.012	0.552 ± 0.028	0.42 ± 0.038^a^
Wild type	0.136 ± 0.009	0.266 ± 0.021	0.131 ± 0.029^d^
N5	0.138 ± 0.004	0.337 ± 0.034	0.199 ± 0.033 ^cd^
N18	0.132 ± 0.004	0.342 ± 0.039	0.21 ± 0.037^c^
N19	0.131 ± 0.005	0.455 ± 0.033	0.324 ± 0.035^b^

Creeping bentgrass plants were cultured in 5 mL liquid ½ MS medium dosed with RDX at 40 mg/L. The masses of wild type and transformants (N5, N18, N19) were measured after 16 days of culture and the biomass gains calculated. The transformed lines N18 and N19 accumulated more biomass than the wild type during the time course. Controls consisted of wild‐type plantlets cultured in MS medium without RDX. Letters indicate biomass gains were significantly different (*P* < 0.05) from other lines (*n* = 3 ± SE).

**Figure 6:**
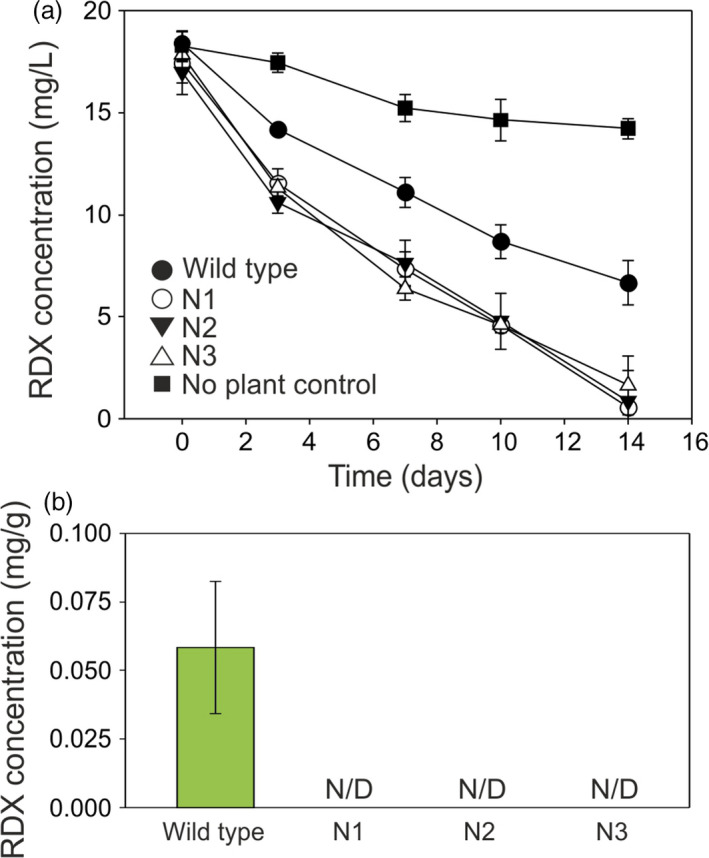
**Uptake of** **RDX** **by** ***xplA*** **‐** ***xplB‐nfsI*** **‐transformed switchgrass grown in liquid culture. (**a) Concentration of RDX in culture medium over the course of the experiment. All three transgenic lines removed RDX from the medium at significantly faster rates than the wild‐type plants (*P* = 0.051, 0.0014 and 0.0016 for lines N1, 2 and 3, respectively, at day 3; *P* = 0.0043 for line N1 at day 7). (b) Concentration of RDX in switchgrass tissue after 14 days (*n* = 3 ± SE, N/D = none detected).

For switchgrass, the course of RDX uptake from the medium is shown in Figure 6a. All three transgenic switchgrass lines removed RDX from the medium at significantly faster rates than the wild‐type plants (*P* = 0.051, 0.0014 and 0.0016 for lines N1, 2 and 3, respectively, at day 3. *P* = 0.0043 for line N1 at day 7). Figure 6b shows that RDX was not detectable in the transgenic switchgrass plants, whereas wild‐type plants contained 0.058 mg RDX per g of leaf tissue. To confirm that the lack of accumulation of RDX in the transgenic switchgrass tissues was caused by degradation, rather than by dilution in growing plant tissue, switchgrass plants were exposed to 20 mg/L RDX in MS medium for 36 h. After this time, leaf tissue from wild‐type plants contained 0.207 ± 0.002 mg/g RDX (*n* = 3 ± SD), whereas RDX was not detected in leaf tissue of transformed switchgrass.

These data demonstrate that all the RDX taken up by transformed switchgrass was degraded within the timescale of the experiment and suggests that RDX degradation is limited by uptake in the transpiration stream. As reported in other studies (Jackson *et al*., 2007; Rylott *et al*., 2006, 2011a; Sabbadin *et al*., 2009), the RDX transformation products MNX and DNX were not detected in either the creeping bentgrass or switchgrass liquid culture studies.

Switchgrass transformants were propagated by inducing cluster shoots from their nodes and the propagated lines tested for RDX uptake from liquid culture. Consistent with the activity determined from the parent transformed plants, the propagated plants had similar rates of RDX removal and degradation (Figure S2), confirming that expression of *xplA* and *xplB* in switchgrass can be transferred during vegetative propagation.

### TNT resistance of transformed grasses

When cultured in ½ MS medium containing 4.5 mg/L TNT both pRCS2‐ABNR‐HR‐transformed line N1 and wild‐type creeping bentgrass plants survived and grew. The ABNR‐HR line removed TNT from the medium more rapidly than the wild type (Figure S3a). While there was no difference in the morphology of the aerial parts of wild‐type or transformed bentgrass, the root morphology was affected by exposure to TNT. The density of mature root hairs was greater for transformed plants compared with that of wild‐type bentgrass after 15 days (Figure S3b and S3c). This result demonstrated that TNT in liquid medium depressed the development of the root system of creeping bentgrass and that the expression of *nfsI* enhanced resistance of plants to TNT. This observation is similar to the finding of TNT toxicity resistance in Arabidopsis transformed with *nfsI* (Hannink *et al*., 2001).

In contrast, we found no difference in TNT resistance between pRCS2‐NABNR‐transformed and wild‐type switchgrass and creeping bentgrass. The root systems of both transformants and wild‐type plants were repressed in MS medium containing 4.5 mg/L TNT. This may be explained by a lack of NR protein production, as the western blot for NR in switchgrass showed only weak bands, although *nfsI* transcript was observed by qRT‐PCR.

### Column studies with switchgrass

**Figure 7:**
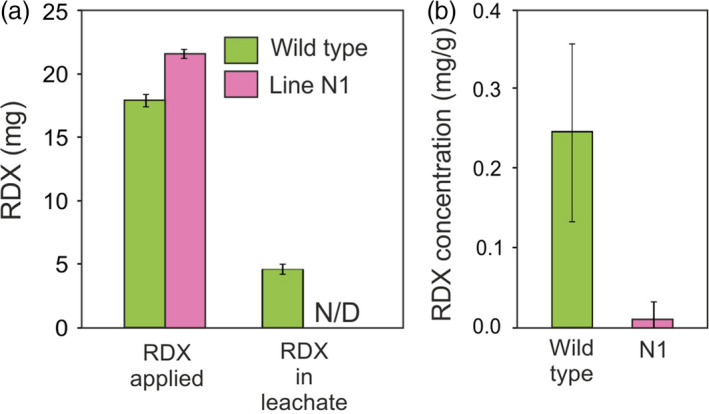
**Recovery of RDX applied to wild‐type and** ***xplA*** **‐** ***xplB‐nfsI*** **‐transformed** switchgrass in column experiments. (a) Mass of RDX applied as solutions containing 30 mg/L, and mass recovered in the leachate by flushing each column with 5 L water. (b) RDX level was significantly less (*P* = 0.0044) in the transgenic leaf tissue compared with the wild‐type leaf tissue in the column experiments after 14 days (*n* = 4 ± SE, N/D = none detected).

To gain an understanding of the ability of the transgenic grass lines to remove RDX from soils on military ranges, column studies containing a sand and gravel mix were conducted. The RDX was applied to the columns containing wild‐type or line N1 switchgrass plants and flushed out three times over the course of 2 months. Following the first two applications, RDX was undetectable in the leachates of columns containing wild‐type and transgenic switchgrass, but wild‐type leaf tissue contained significantly more RDX than transgenic leaf tissue (data not shown). The results of the third application of RDX are shown in Figure 7a. About one‐fourth of the applied RDX was recovered in the leachate from the wild‐type columns, whereas RDX was not detected in the leachate from the transgenic columns. In the leaf tissues, RDX level was significantly less (*P* = 0.0044) in the transgenic leaf tissue compared with the wild‐type tissue (Figure 7b).

## Discussion

The use of transgenic plants has been proposed for the phytoremediation of pollutants including metals, explosives, petroleum, solvents and polycyclic aromatic hydrocarbons (Bizily *et al*., 2000; Chen *et al*., 2010; Doty *et al*., 2000, 2007; Karavangeli *et al*., 2005; Rylott *et al*., 2015) and demonstrated in tobacco, Arabidopsis and *Populus*. For application to soil remediation, species such as grasses are desirable, but transformation of grasses with phytoremediation genes has not been demonstrated. This work focuses on the development of creeping bentgrass and switchgrass lines transformed with *xplA, xplB* and *nfsI* for the degradation of RDX and detoxification of TNT on live‐fire training ranges. These perennial grasses provide year‐round cover, are adaptable to different environmental conditions on training ranges and have wide geographic range. They also have highly dense and deep rooting systems which provide accessibility to and uptake of RDX and TNT. In addition, these species provide good erosion control, nesting and invertebrate habitats. Together, the studies here show that these transformed species can take up and degrade RDX more efficiently than wild‐type plants.

These results demonstrate that compared with transformed creeping bentgrass, transformed switchgrass degraded RDX more efficiently. The RDX remained at a detectable concentration in most transformed lines of creeping bentgrass, while RDX was not detected in any of the four transformed lines of switchgrass examined. This high activity may have been due to the promoter used to drive the expression of *xplA*, the ubiquitin promoter, which was cloned from switchgrass (Mann *et al*., 2011).

Once RDX enters the plant roots, it is transported to leaf tissue in the transpiration stream. Studies in poplar tissues (*Populus deltoides* x *nigra* DN‐34) show that RDX in leaf tissue is partially reduced to MNX and DNX. (Van Aken *et al*., 2004). In agreement with other studies (Jackson *et al*., 2007; Rylott *et al*., 2006, 2011a,b; Sabbadin *et al*., 2009), these metabolites were not detected in the liquid culture studies reported here and it is likely that that these compounds are either produced at very low levels or rapidly conjugated by the plant.

Despite high uptake and translocation rates, the ability of wild‐type plants to degrade RDX is low (Just and Schnoor, 2004; Winfield *et al*., 2004). Thus, when plants are grown in RDX‐contaminated media, RDX accumulates in the leaf tissue, limiting the capacity of the plant to remove further RDX from the soil. As shown here, the accumulation of RDX in the leaf tissue of wild‐type creeping bentgrass also suppresses plant growth (Table 1). Not only is the RDX‐degrading ability of plants inherently low, RDX accumulated in plant tissue is likely to re‐enter the soil and potentially leach to groundwater following plant senescence. These factors limit the usefulness of wild‐type plants for the phytoremediation of RDX in training ranges and provide an explanation for the persistent pollution of groundwater under vegetated training ranges. The results of the column studies with wild‐type switchgrass are consistent with these findings, showing that wild‐type plants were initially able to stop RDX leaching, but that RDX accumulated in the wild‐type leaves, and uptake by wild‐type plants subsequently declined.

Although transformation of creeping bentgrass with the *nfsI*‐containing vector pRCS‐ABNR‐HR conferred increased TNT resistance, the level of resistance was poor when compared to the performance of *nfs‐*transformed tobacco and Arabidopsis in other studies (Hannink *et al*., 2001; Rylott *et al*., 2011a). Furthermore, switchgrass transformed with the *nfsI*‐containing vector pRCS2‐NABNR did not have increased resistance to TNT compared with the wild type. The results presented here indicate that the lack of TNT resistance in the *nfsI*‐transformed grass species is due to low transcription *nfsI*, and possibly due to poor performance of the 35S promoter in these monocot species (McElroy *et al*., 1990). Another explanation for the lack of increased resistance is that the *xplB* and *nfsI* expression cassettes were transcribed in opposite directions in the pRCS2‐NABNR vector, possibly yielding antisense RNA by read‐through transcription, which triggered silencing (Kooter *et al*., 1999). In future work, the 35S promoter in pRCS2‐NABNR will be replaced with a monocot‐specific promoter to enhance the expression level of *nfsI* and to optimize the transcription direction of different cassettes. These studies underline the importance of promoter choice in monocot transformation studies. In recent years, several constitutive promoters cloned from monocot plant species have been shown to drive high expression of reporter genes in monocot hosts (Kamo, 2003; Park *et al*., 2010). Alternatively, there have been reports of virus promoters successfully used for foreign gene expression in monocot plant species (Schenk *et al*., 1999, 2001).

Rather than using a bacterial nitroreductase transgene to confer enhanced resistance to TNT phytotoxicity, there are alternative approaches. As with RDX, plants have only a limited ability to detoxify TNT, the elucidation of these pathways in Arabidopsis has shown that OPRs, UGTs and GSTs are all involved and that overexpression of these TNT detoxification encoding genes can significantly enhance tolerance of Arabidopsis to TNT (Beynon *et al*., 2009; Gandia‐Herrero *et al*., 2008; Gunning *et al*., 2014). Furthermore, a recent study has shown that the mutation of monodehydroascorbate reductase 6 in Arabidopsis greatly enhanced TNT tolerance (Johnston *et al*., 2015), a finding that could perhaps be applied into other species using nontransgenic gene editing techniques.

In conclusion, this is the first report of genetically transformed grasses for the phytoremediation of the explosive and environmental pollutant, RDX. Creeping bentgrass and switchgrass were successfully transformed with the bacterial genes to confer RDX degradation, *xplA* and *xplB*. Both transformed grasses were able to degrade RDX at substantially higher rates than untransformed plants, in the best‐performing lines preventing the accumulation of RDX in the plant tissues. The use of these plants is a promising biotechnology to prevent contamination of groundwater under live‐fire training ranges by degrading RDX taken up from the root zone.

## Experimental procedures

### Plant materials, explant sterilization and callus induction

A commercial lowland switchgrass cultivar, Alamo, was used for this study. Mature seeds of Alamo were surface‐sterilized in 20% bleach for 30 min, rinsed three times with sterile water and left overnight in the dark at 24 °C. On the second day, the sterilization procedure was repeated as described above. Embryogenic callus induction, infection and selection of transformed switchgrass plantlets were carried out as described in Li and Qu (2011). The method for callus induction and transformation of creeping bentgrass followed the previous protocol of Lee *et al*. (2011).

### Plasmid construction and transformation protocol

The 35S cassette was released from pSAT4a (Chung *et al*., 2005) as an *Age*I‐*Not*I fragment and used to replace the ocs expression cassette in pSAT1a to produce pSAT1a‐35S. The hygromycin resistance gene, *hygromycin B phosphotransferase* (*hpt*), was cloned by PCR from pcambia1301 and inserted into pSAT1a‐35S to produce pSAT1a‐35S‐*hpt*. All the primers used in this study are shown in Table S1. The 35S‐*hpt*‐35ST expression cassette was released from pSAT1a‐35S‐*hpt* as an *Asc*I fragment and inserted into the binary vector pPZP‐RCS2 to produce prcs2‐35S‐*hpt*. The *xplA*,* xplB* and *nfsI* genes were cloned by PCR from the vectors pMLBart‐*xplA*, pART27‐*xplB* and pART27‐*nfsI* (Rylott *et al*., 2011a), using AccuPrime Taq DNA polymerase high fidelity (Invitrogen) for amplification. The *xplA* gene was inserted into the pSAT6a vector to produce pSAT6a‐*xplA*. The expression cassette rbc‐*xplA*‐rbc T was released as a PI‐*Psp*I fragment from pSAT6a‐*xplA* and inserted into the binary vector pRCS2‐35S‐*hpt* to produce pRCS2‐6*xplA*. The *xplB* gene was inserted into pSAT4a to produce pSAT4a‐*xplB*, and the expression cassette 35S‐*xplB*‐35ST was released from pSAT4a‐*xplB* as an I‐*Sce*I fragment and inserted into pRCS2‐*xplA* to produce prcs2‐6*xplA*‐4*xplB*. The *nfsI* gene was cloned and inserted into pSAT7a to produce pSAT7a‐*nfsI*, and the expression cassette act‐*nfsI*‐agsT was released from pSAT7a‐*nfsI* as a PI‐*Tli*I fragment and inserted into prcs2‐6*xplA*‐4*xplB* to produce pRCS2‐ABNR‐HR.

To enhance the expression level of transgenes in switchgrass, three monocot‐specific promoters were cloned from the pANIC vector system (Mann *et al*., 2012) by PCR and used to replace the promoters in the pSAT vectors to produce a new set of vectors, which were designated the pNSAT vectors. The actin promoter from rice (*Oryza sativa*) and the ubiquitin promoters from corn (*Zea mays*) and switchgrass (*Panicum virgatum*) were cloned by PCR using the pANIC vector as a template, replacing the promoters in pSAT1a‐35S, pSAT3a and pSAT6a, respectively, to produce the pNSAT1a, pNSAT3a and pNSAT6a cloning vectors. The *hpt, xplA, xplB* and *nfsI* genes were inserted into pNSAT1a, pNSAT6a, pNSAT3a and pSAT4a, respectively, and the expression cassettes of these genes were inserted into the pRCS2 binary vector to produce pRCS2‐NABNR.

The binary vectors pRCS2‐ABNR‐HR and pRCS2‐NABNR were transferred into *Agrobacterium* strain EHA105 by the freeze–thaw method (Chen *et al*., 1994) and the resulting strain, EHA105 (pRCS2‐ABNRHR/pRCS2‐NABNR), was grown in LB medium with 50 m/L rifampicin, 100 mg/L spectinomycin and 300 mg/L streptomycin for infection of the embryogenic callus of switchgrass and creeping bentgrass.

### Molecular analysis of transgenic plants

For PCR analysis, the DNeasy plant mini kit (Qiagen, Valencia, CA) was used to purify DNA from hygromycin‐resistant plants. The PCRs were carried out by amplifying the expression cassette region of the *xplB* gene, including parts of the promoter and terminator sequences (Table S1).

For transcript analysis, mRNA was extracted from mature creeping bentgrass, switchgrass leaf blades and Arabidopsis 6‐week‐old rosette leaves using the Isolate II RNA Plant Kit (Bioline). Five micrograms of total RNA was used to synthesize cDNA using oligo (dT) 12‐18 primers (Invitrogen) and SuperScript III Reverse Transcriptase (Invitrogen). Quantitative real‐time PCR (qPCR) was performed using a StepOne Plus real‐time PCR detection system with SYBR green (Applied Biosystems). Bentgrass values were normalized to the 5.8S gene. Switchgrass values were normalized to the switchgrass reference gene eIF‐4a (Gimeno *et al*., 2014; GenBank accession number GR877213). Primers sequences for *ACT2*,* xplA*,* xplB* and *nfsI* were as reported previously (Rylott *et al*., 2011a). Transcript abundance was expressed relative to the levels of the *xplA*‐*xplB*‐*nfsI* expressing Arabidopsis line 7D (Rylott *et al*., 2011a).

### Protein extraction and immunoblot analyses

For protein expression analysis, eight micrograms of crude protein extract from leaf tissues was loaded per lane. Antibodies were used as reported previously, XplA (Rylott *et al*., 2006), XplB (Jackson *et al*., 2007) and NR (Rylott *et al*., 2011a). Three replicate blots were made for each protein and band intensities quantified from pixel measurements of western blot images using ImageJ software.

### RDX uptake by transformed switchgrass and creeping bentgrass

Wild‐type and transformed grass plants with similar biomass and at the same development status were selected and cultured in 5 mL ½ MS media without sugar and supplied with RDX at 40 mg/L for creeping bentgrass and 20 mg/L for switchgrass under 16‐h light, 8‐h dark photoperiod at 25 °C for 15 days. The concentration of RDX in the medium was assayed at regular time intervals. The volume of medium was refilled back to 5 mL with water every time before sampling. After 15 days of culture, the RDX concentration in plant tissue was also analysed. Plant tissues (100 mg) were collected and freeze‐dried using a Labconco Freezone 4.5 Liter Freeze Dry System (Labconco, Kansas) and ground to powder using a Fast Prep 24 (MP Biomedicals, LLC., Solon). The plant tissue powders were immersed in 1 mL methanol and incubated for 12 h at room temperature with shaking. The tubes were then centrifuged twice at 15 871 ***g*** for 10 min. The supernatant (800 μL) was collected for HPLC analysis.

### HPLC quantification of aqueous RDX

RDX concentrations in culture media were analysed with a modular Waters HPLC system consisting of a Waters 717 autosampler, two Waters 515 HPLC pumps and a Waters 2996 photodiode array detector. A 4.6 by 250 mm Waters C18 column was used for separation under conditions similar to those outlined previously (Andeer *et al*., 2013), with concentration determined based on absorbance at 240 nm. Peak integrations and analyses were conducted using Millennium 32 software (Waters, Milford, MA). The limit of detection of RDX by this method is 0.01 mg/L.

### TNT uptake by transformed creeping bentgrass

Wild‐type and transformed creeping bentgrass plantlets were cultured in 30 mL liquid ½ MS medium amended with TNT at 4.5 mg/L in flasks at 20 °C with shaking. The light intensity is at 13.875 μmol/m^2^·s. Each flask contained three independent creeping bentgrass plantlets with biomass of about 100 mg, and each treatment was repeated four times. The growth of the roots was observed and the root hairs were photographed after 15 days of culture.

### Column studies

Twelve polyvinyl chloride (PVC) columns were constructed with PVC tubing (90 mm diameter, 0.5 m long). Media for the columns were a mix of 75% gravel and 25% sand.

Eight matching columns were planted, four each, with wild‐type and transgenic grasses and the grasses were grown to over 0.5 m and pruned back to about 0.5 m uniform height. Then, the columns were dosed with equal amounts of RDX on the first, third and fifth day of the first week, and, as needed, again on the following week on the same schedule. The RDX was dosed using with aliquots of 125 mL of RDX solution containing approximately 7.5 mg RDX. The void volume of the planted columns was approximately 1.5 L. Following each dosing, the planted columns were incubated for 1 week with 125 mL 1× Hoagland's medium. Two days after the final RDX dosing, the planted columns were flushed with 5 L DI water and the effluent was collected in 500 mL aliquots, which were sampled for analysis of RDX. A total of 5 L DI water was used to flush the columns clean of RDX, until RDX was undetectable by HPLC, usually 3.5–4.5 L.

### Statistical analysis

Data were analysed for statistical significance using ANOVA (Microsoft Excel 2016). When ANOVA gave a significant difference, Fisher's least significant difference (LSD) method was performed to compare the means and the statistical groupings are indicated by labels in the figures.

## Supporting information


**Figure S1.** Molecular characterization of transgene creeping bentgrass.

**Figure S2.** Uptake of RDX by propagated plants from *xplA*‐*xplB‐nfsI* transformed switchgrass grown in liquid culture.

**Figure S3.** Studies on liquid‐culture grown *xplA*‐*xplB‐nfsI* transformed creeping bentgrass exposed to TNT.

**Table S1.** The DNA sequences of primers used in this study.
**Data S1.** Methods

## References

[pbi12661-bib-0001] Andeer, P. , Stahl, D.A. , Lillis, L. and Strand, S.E. (2013) Identification of microbial populations assimilating nitrogen from RDX in munitions contaminated military training range soils by high sensitivity stable isotope probing. Environ. Sci. Technol. 47, 10356–10363. 23909596 10.1021/es401729c

[pbi12661-bib-0002] Beynon, E.R. , Symons, Z.C. , Jackson, R.G. , Lorenz, A. , Rylott, E.L. and Bruce, N.C. (2009) The role of oxophytodienoate reductases in the detoxification of the explosive 2,4,6‐trinitrotoluene by Arabidopsis. Plant Physiol. 151, 253–261. 19605548 10.1104/pp.109.141598 PMC2735992

[pbi12661-bib-0003] Bizily, S.P. , Rugh, C.L. and Meagher, R.B. (2000) Phytodetoxification of hazardous organomercurials by genetically engineered plants. Nat. Biotechnol. 18, 213–217. 10657131 10.1038/72678

[pbi12661-bib-0004] Brentner, L.B. , Mukherji, S.T. , Walsh, S.A. and Schnoor, J.L. (2010) Localization of hexahydro‐1,3,5‐trinitro‐1,3,5‐triazine (RDX) and 2,4,6‐trinitrotoluene (TNT) in poplar and switchgrass plants using phosphor imager autoradiography. Environ. Pollut. 158, 470–475. 19782446 10.1016/j.envpol.2009.08.022

[pbi12661-bib-0005] Bryant, C. and DeLuca, M. (1991) Purification and characterization of an oxygen‐insensitive NAD(P)H nitroreductase from *Enterobacter cloacae* . J. Biol. Chem. 266, 4119–4125. 1999405

[pbi12661-bib-0006] Burris, J.N. , Mann, D.G.J. , Joyce, B.L. and Stewart, C.N. (2009) An improved tissue culture system for embryogenic callus production and plant regeneration in switchgrass (*Panicum virgatum* L.). Bioenerg. Res. 2, 267–274.

[pbi12661-bib-0007] Chen, H. , Nelson, R.S. and Sherwood, J.L. (1994) Enhanced recovery of transformants of *Agrobacterium tumefaciens* after freeze‐thaw transformation and drug selection. Biotechniques, 16, 670. 8024787

[pbi12661-bib-0008] Chen, L.M. , Yurimoto, H. , Li, K.Z. , Orita, I. , Akita, M. , Kato, N. , Sakai, Y. *et al*. (2010) Assimilation of formaldehyde in transgenic plants due to the introduction of the bacterial ribulose monophosphate pathway genes. Biosci. Biotechnol. Biochem. 74, 627–635. 20208346 10.1271/bbb.90847

[pbi12661-bib-0009] Chung, S.M. , Frankman, E.L. and Tzfira, T. (2005) A versatile vector system for multiple gene expression in plants. Trends Plant Sci. 10, 357–361. 15993643 10.1016/j.tplants.2005.06.001

[pbi12661-bib-0010] Cornejo, M.J. , Luth, D. , Blankenship, K.M. , Anderson, O.D. and Blechl, A.E. (1993) Activity of a maize ubiquitin promoter in transgenic rice. Plant Mol. Biol. 23, 567–581. 8219091 10.1007/BF00019304

[pbi12661-bib-0011] Deng, Y. , Ai, J. , Guan, X. , Wang, Z. , Yan, B. , Zhang, D. , Liu, C. *et al*. (2014) MicroRNA and messenger RNA profiling reveals new biomarkers and mechanisms for RDX induced neurotoxicity. BMC Genom. 15(Suppl 11), S1. 10.1186/1471-2164-15-S11-S1 PMC4304176 25559034

[pbi12661-bib-0012] Doty, S.L. , Shang, T.Q. , Wilson, A.M. , Tangen, J. , Westergreen, A.D. , Newman, L.A. , Strand, S.E. *et al*. (2000) Enhanced metabolism of halogenated hydrocarbons in transgenic plants containing mammalian cytochrome P450 2E1. Proc. Natl Acad. Sci. USA, 97, 6287–6291. 10841534 10.1073/pnas.97.12.6287 PMC18595

[pbi12661-bib-0013] Doty, S.L. , James, C.A. , Moore, A.L. , Vajzovic, A. , Singleton, G.L. , Ma, C. , Khan, Z. *et al*. (2007) Enhanced phytoremediation of volatile environmental pollutants with transgenic trees. Proc. Natl Acad. Sci. USA, 104, 16816–16821. 17940038 10.1073/pnas.0703276104 PMC2040402

[pbi12661-bib-0014] Gandia‐Herrero, F. , Lorenz, A. , Larson, T. , Graham, I.A. , Bowles, D.J. , Rylott, E.L. and Bruce, N.C. (2008) Detoxification of the explosive 2,4,6‐trinitrotoluene in Arabidopsis: discovery of bifunctional O‐ and C‐glucosyltransferases. Plant J. 56, 963–974. 18702669 10.1111/j.1365-313X.2008.03653.x

[pbi12661-bib-0015] Gimeno, J. , Eattock, N. , Van Deynze, A. and Blumwald, E. (2014) Selection and validation of reference genes for gene expression analysis in switchgrass (*Panicum virgatum)* using quantitative real‐time RT‐PCR. PLoS ONE, 9, e91474. 24621568 10.1371/journal.pone.0091474 PMC3951385

[pbi12661-bib-0016] Gunning, V. , Tzafestas, K. , Sparrow, H. , Johnston, E.J. , Brentnall, A.S. , Potts, J.R. , Rylott, E.L. *et al*. (2014) Arabidopsis glutathione transferases U24 and U25 exhibit a range of detoxification activities with the environmental pollutant and explosive, 2,4,6‐trinitrotoluene. Plant Physiol. 165, 854–865. 24733884 10.1104/pp.114.237180 PMC4044842

[pbi12661-bib-0017] Hannink, N. , Rosser, S.J. , French, C.E. , Basran, A. , Murray, J.A. , Nicklin, S. and Bruce, N.C. (2001) Phytodetoxification of TNT by transgenic plants expressing a bacterial nitroreductase. Nat. Biotechnol. 19, 1168–1172. 11731787 10.1038/nbt1201-1168

[pbi12661-bib-0018] Jackson, R.G. , Rylott, E.L. , Fournier, D. , Hawari, J. and Bruce, N.C. (2007) Exploring the biochemical properties and remediation applications of the unusual explosive‐degrading P450 system XplA/B. Proc. Natl Acad. Sci. USA, 104, 16822–16827. 17940033 10.1073/pnas.0705110104 PMC2040458

[pbi12661-bib-0019] Johnston, E.J. , Rylott, E.L. , Beynon, E. , Lorenz, A. , Chechik, V. and Bruce, N.C. (2015) Monodehydroascorbate reductase mediates TNT toxicity in plants. Science, 349, 1072–1075. 26339024 10.1126/science.aab3472

[pbi12661-bib-0020] Just, C.L. and Schnoor, J.L. (2004) Phytophotolysis of hexahydro‐1,3,5‐trinitro‐1,3,5‐triazine (RDX) in leaves of reed canary grass. Environ. Sci. Technol. 38, 290–295. 14740749 10.1021/es034744z

[pbi12661-bib-0021] Kamo, K.K. (2003) Long‐term expression of the *uidA* gene in *Gladiolus* plants under control of either the ubiquitin, rolD, mannopine synthase, or cauliflower mosaic virus promoters following three seasons of dormancy. Plant Cell Rep. 21, 797–803. 12789525 10.1007/s00299-003-0578-9

[pbi12661-bib-0022] Karavangeli, M. , Labrou, N.E. , Clonis, Y.D. and Tsaftaris, A. (2005) Development of transgenic tobacco plants overexpressing maize glutathione S‐transferase I for chloroacetanilide herbicides phytoremediation. Biomol. Eng. 22, 121–128. 16085457 10.1016/j.bioeng.2005.03.001

[pbi12661-bib-0023] Kooter, J.M. , Matzke, M.A. and Meyer, P. (1999) Listening to the silent genes: transgene silencing, gene regulation and pathogen control. Trends Plant Sci. 4, 340–347. 10462766 10.1016/s1360-1385(99)01467-3

[pbi12661-bib-0024] Lee, K.W. , Kim, K.Y. , Kim, K.H. , Lee, B.H. , Kim, J.S. and Lee, S.H. (2011) Development of antibiotic marker‐free creeping bentgrass resistance against herbicides. Acta Biochim. Biophys. Sin. 43, 13–18. 21173055 10.1093/abbs/gmq106

[pbi12661-bib-0025] Li, R.Y. and Qu, R.D. (2011) High throughput Agrobacterium‐mediated switchgrass transformation. Biomass Bioenerg, 35, 1046–1054.

[pbi12661-bib-0026] Mann, D.G. , King, Z.R. , Liu, W. , Joyce, B.L. , Percifield, R.J. , Hawkins, J.S. , LaFayette, P.R. *et al*. (2011) Switchgrass (*Panicum virgatum* L.) polyubiquitin gene (PvUbi1 and PvUbi2) promoters for use in plant transformation. BMC Biotechnol. 11, 74. 21745390 10.1186/1472-6750-11-74 PMC3161867

[pbi12661-bib-0027] Mann, D.G. , Lafayette, P.R. , Abercrombie, L.L. , King, Z.R. , Mazarei, M. , Halter, M.C. , Poovaiah, C.R. *et al*. (2012) Gateway‐compatible vectors for high‐throughput gene functional analysis in switchgrass (*Panicum virgatum* L.) and other monocot species. Plant Biotechnol. J. 10, 226–236. 21955653 10.1111/j.1467-7652.2011.00658.x

[pbi12661-bib-0028] McElroy, D. , Zhang, W. , Cao, J. and Wu, R. (1990) Isolation of an efficient actin promoter for use in rice transformation. Plant Cell, 2, 163–171. 2136633 10.1105/tpc.2.2.163 PMC159873

[pbi12661-bib-0029] Palazzo, A.J. , Jensen, K.B. , Waldron, B.L. and Cary, T.J. (2005) Effects of tank tracking on range grasses. J. Terramech. 42, 177–191.

[pbi12661-bib-0030] Park, S.H. , Yi, N. , Kim, Y.S. , Jeong, M.H. , Bang, S.W. , Choi, Y.D. and Kim, J.K. (2010) Analysis of five novel putative constitutive gene promoters in transgenic rice plants. J. Exp. Bot. 61, 2459–2467. 20363869 10.1093/jxb/erq076 PMC2877896

[pbi12661-bib-0031] Ramamoorthy, R. and Kumar, P.P. (2012) A simplified protocol for genetic transformation of switchgrass (*Panicum virgatum* L.). Plant Cell Rep. 31, 1923–1931. 22733209 10.1007/s00299-012-1305-1

[pbi12661-bib-0032] Rivera, R. , Medina, V.F. , Larson, S.L. and McCutcheon, S.C. (1998) Phytotreatment of TNT‐contaminated groundwater. J. Soil. Contam. 7, 511–529.

[pbi12661-bib-0033] Rylott, E.L. and Bruce, N.C. (2009) Plants disarm soil: engineering plants for the phytoremediation of explosives. Trends Biotechnol. 27, 73–81. 19110329 10.1016/j.tibtech.2008.11.001

[pbi12661-bib-0034] Rylott, E.L. , Jackson, R.G. , Edwards, J. , Womack, G.L. , Seth‐Smith, H.M. , Rathbone, D.A. , Strand, S.E. *et al*. (2006) An explosive‐degrading cytochrome P450 activity and its targeted application for the phytoremediation of RDX. Nat. Biotechnol. 24, 216–219. 16429147 10.1038/nbt1184

[pbi12661-bib-0035] Rylott, E.L. , Budarina, M.V. , Barker, A. , Lorenz, A. , Strand, S.E. and Bruce, N.C. (2011a) Engineering plants for the phytoremediation of RDX in the presence of the co‐contaminating explosive TNT. New Phytol. 192, 405–413. 21729248 10.1111/j.1469-8137.2011.03807.x

[pbi12661-bib-0036] Rylott, E.L. , Jackson, R.G. , Sabbadin, F. , Seth‐Smith, H.M. , Edwards, J. , Chong, C.S. , Strand, S.E. *et al*. (2011b) The explosive‐degrading cytochrome P450 XplA: biochemistry, structural features and prospects for bioremediation. Biochim. Biophys. Acta, 1814, 230–236. 20624490 10.1016/j.bbapap.2010.07.004

[pbi12661-bib-0037] Rylott, E.L. , Johnston, E.J. and Bruce, N.C. (2015) Harnessing microbial gene pools to remediate persistent organic pollutants using genetically modified plants‐ a viable technology? J. Exp. Bot. 66, 6519–6533. 26283045 10.1093/jxb/erv384

[pbi12661-bib-0038] Sabbadin, F. , Jackson, R. , Haider, K. , Tampi, G. , Turkenburg, J.P. , Hart, S. , Bruce, N.C. *et al*. (2009) The 1.5‐A structure of XplA‐heme, an unusual cytochrome P450 heme domain that catalyzes reductive biotransformation of royal demolition explosive. J. Biol. Chem. 284, 28467–28475. 19692330 10.1074/jbc.M109.031559 PMC2788895

[pbi12661-bib-0039] Schenk, P.M. , Sagi, L. , Remans, T. , Dietzgen, R.G. , Bernard, M.J. , Graham, M.W. and Manners, J.M. (1999) A promoter from sugarcane bacilliform badnavirus drives transgene expression in banana and other monocot and dicot plants. Plant Mol. Biol. 39, 1221–1230. 10380808 10.1023/a:1006125229477

[pbi12661-bib-0040] Schenk, P.M. , Remans, T. , Sagi, L. , Elliott, A.R. , Dietzgen, R.G. , Swennen, R. , Ebert, P.R. *et al*. (2001) Promoters for pregenomic RNA of banana streak badnavirus are active for transgene expression in monocot and dicot plants. Plant Mol. Biol. 47, 399–412. 11587511 10.1023/a:1011680008868

[pbi12661-bib-0041] Seth‐Smith, H.M. , Rosser, S.J. , Basran, A. , Travis, E.R. , Dabbs, E.R. , Nicklin, S. and Bruce, N.C. (2002) Cloning, sequencing, and characterization of the hexahydro‐1,3,5‐trinitro‐1,3,5‐triazine degradation gene cluster from *Rhodococcus rhodochrous* . Appl. Environ. Microbiol. 68, 4764–4771. 12324318 10.1128/AEM.68.10.4764-4771.2002 PMC126434

[pbi12661-bib-0042] Van Aken, B. , Yoon, J.M. , Just, C.L. and Schnoor, J.L. (2004) Metabolism and mineralization of hexahydro‐1,3,5‐trinitro‐1,3,5‐triazine inside poplar tissues (*Populus deltoides* x *nigra* DN‐34). Environ. Sci. Technol. 38, 4572–4579. 15461165 10.1021/es049837a

[pbi12661-bib-0043] Winfield, L.E. , Rodger, J.H. and D'surney, S.J. (2004) The responses of selected terrestrial plants to short (<12 days) and long term (2, 4 and 6 weeks) hexahydro‐1,3,5‐trinitro‐1,3,5‐triazine (RDX) exposure. Part I: growth and developmental effects. Ecotoxicology, 13, 335–347. 15344514 10.1023/b:ectx.0000033091.78180.3d

[pbi12661-bib-0044] Xi, Y.J. , Fu, C.X. , Ge, Y.X. , Nandakumar, R. , Hisano, H. , Bouton, J. and Wang, Z.Y. (2009) Agrobacterium‐mediated transformation of switchgrass and inheritance of the transgenes. Bioenerg. Res. 2, 275–283.

[pbi12661-bib-0045] Xiong, R.C. , Fern, J.T. , Keffer, D.J. , Fuentes‐Cabrera, M. and Nicholson, D.M. (2009) Molecular simulations of adsorption and diffusion of RDX in IRMOF‐1. Mol. Simulat. 35, 910–919.

[pbi12661-bib-0046] Zhou, M. , Li, D. , Li, Z. , Hu, Q. , Yang, C. , Zhu, L. and Luo, H. (2013) Constitutive expression of a miR319 gene alters plant development and enhances salt and drought tolerance in transgenic creeping bentgrass. Plant Physiol. 161, 1375–1391. 23292790 10.1104/pp.112.208702 PMC3585603

