# Supplementary figures and images for: Expression in grasses of multiple transgenes for degradation of munitions compounds on live‐fire training ranges
Source: Plant Biotechnol J. 2016 Dec 29;15(5):624–33. doi: 10.1111/pbi.12661 (PMC5399000; doi:10.1111/pbi.12661)

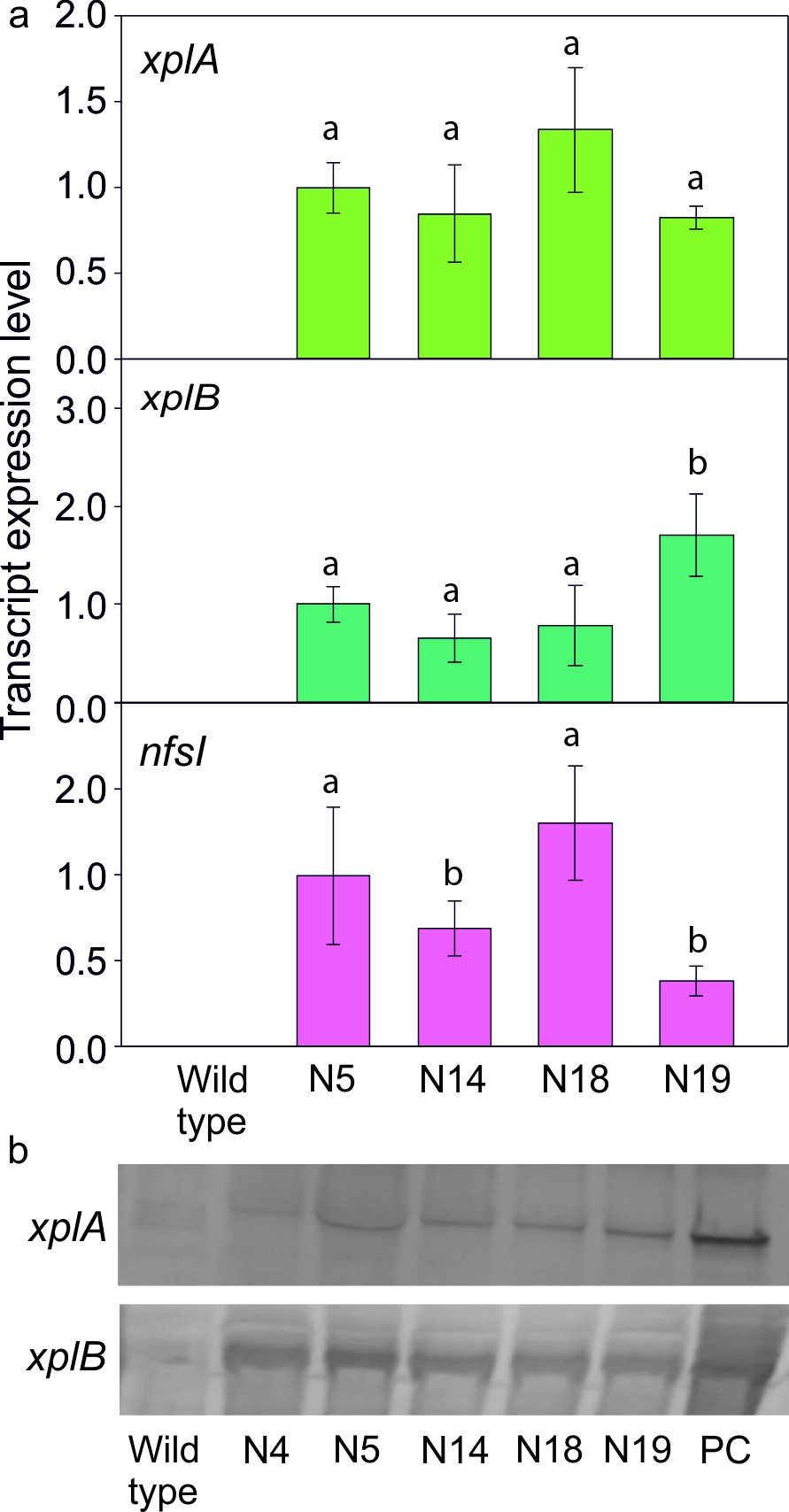

Supplement: Supplementary file 1 — Figure S1. Molecular characterization of transgene creeping bentgrass. [file PBI-15-624-s003.jpg]

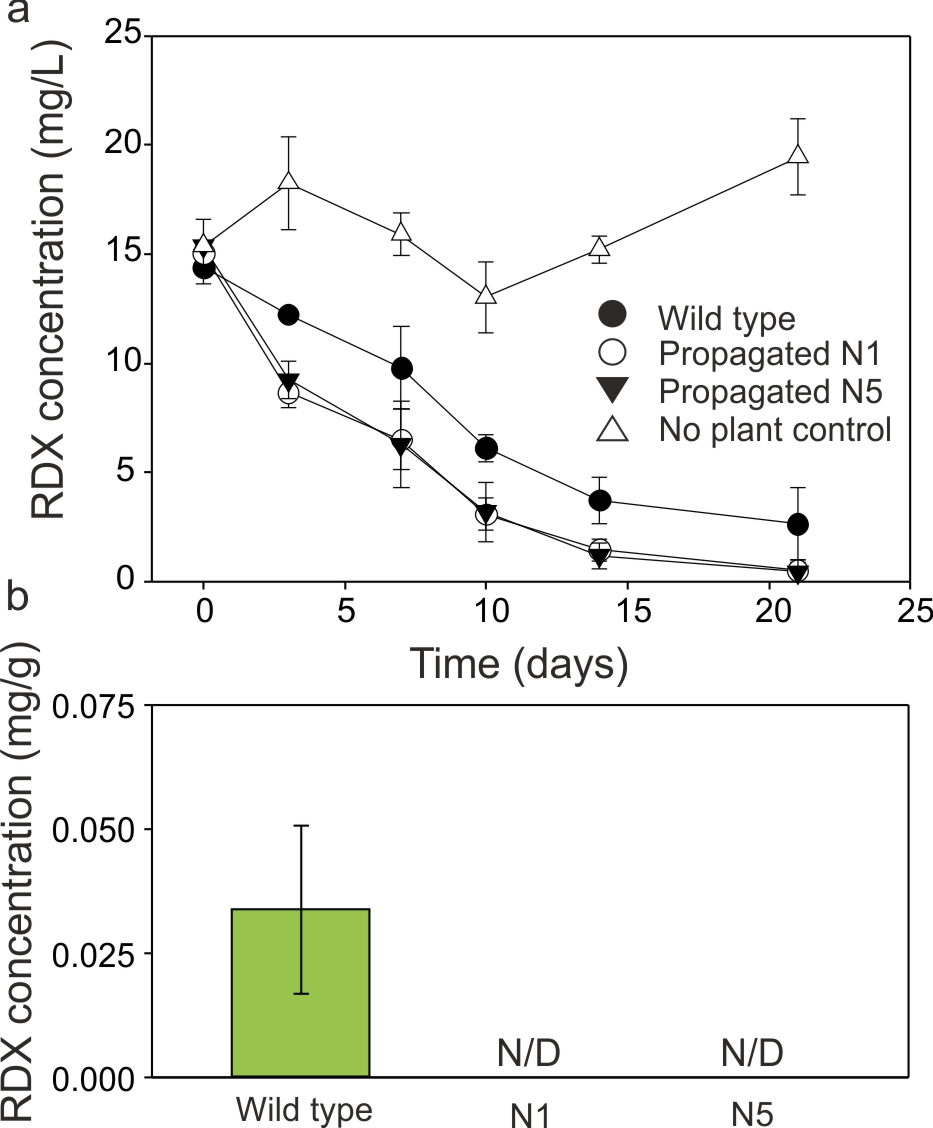

Supplement: Supplementary file 2 — Figure S2. Uptake of RDX by propagated plants from xplA‐xplB‐nfsI transformed switchgrass grown in liquid culture. [file PBI-15-624-s004.jpg]

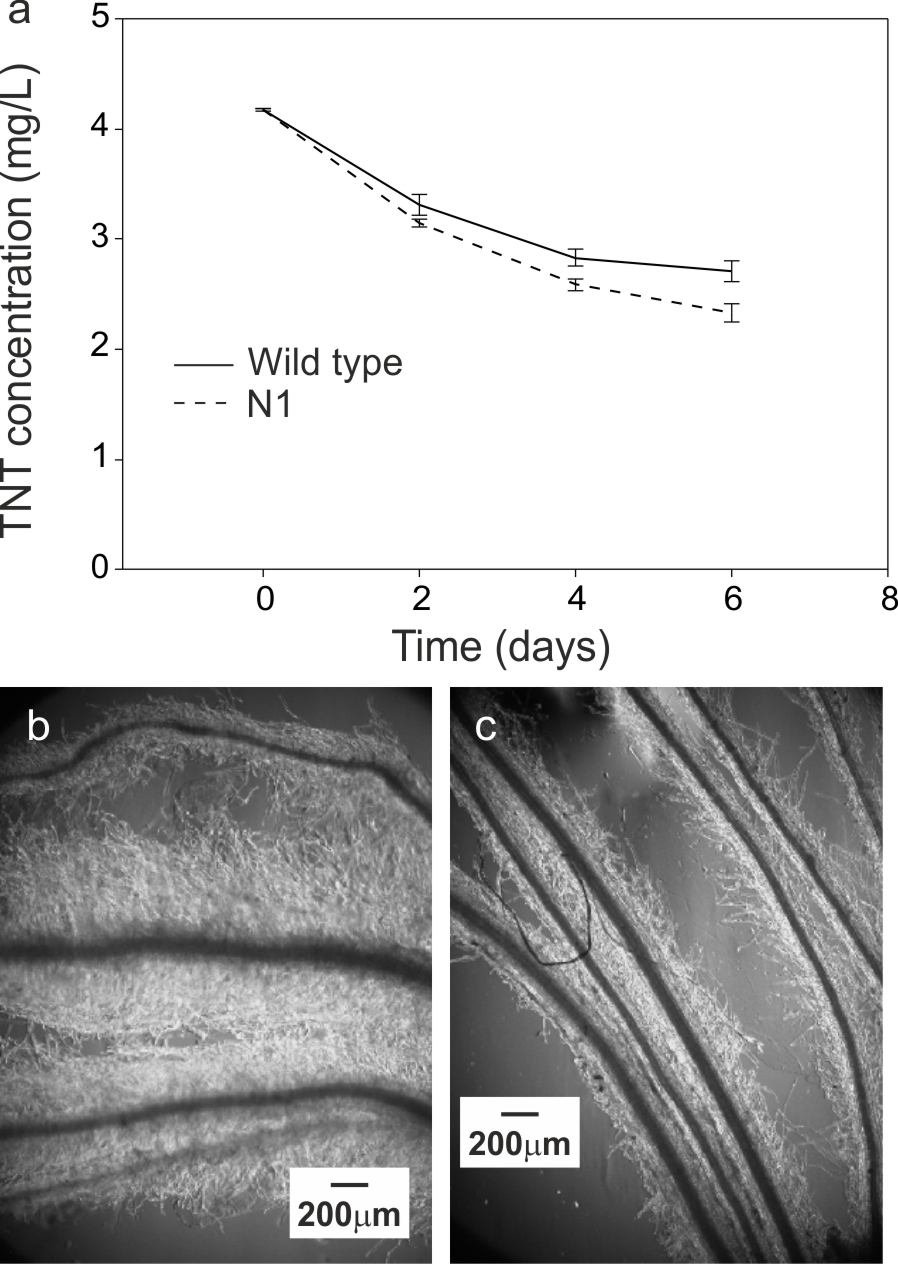

Supplement: Supplementary file 3 — Figure S3. Studies on liquid‐culture grown xplA‐xplB‐nfsI transformed creeping bentgrass exposed to TNT. [file PBI-15-624-s002.jpg]
