# Supplementary material for: Expression in grasses of multiple transgenes for degradation of munitions compounds on live‐fire training ranges
Source: Plant Biotechnol J. 2016 Dec 29;15(5):624–33. doi: 10.1111/pbi.12661 (PMC5399000; doi:10.1111/pbi.12661)
Supplement: Supplementary file 4 — Table S1. The DNA sequences of primers used in this study. Data S1. Methods [file PBI-15-624-s001.docx]

# Supplementary Materials

**Supplementary Table 1.** The DNA sequences of primers used in this study.

| **Primer name** | **Sequence (5’ - 3’)** | **Flanking Restriction site** | **Details** |
| --- | --- | --- | --- |
| HptF3 | TTGAATTCATTATGAAAAAGCCTGAACTC | EcoRI | Clone and Insert *hpt* gene into pSAT1a-35S to produce pSAT35S-*hpt* |
| HptR3 | ATTGGATCCCTATTTCTTTGCC | BamHI |  |
| XplaF5 | ttAAGCTTACCATGGccgacgtaactgtcctg | HindIII | Clone and insert *xplA* gene into pSAT6a and pnSAT6a |
| XplaR5 | TTAAGCTTTCAGGACAGGACGATCGGC | HindIII |  |
| XplbF1 | ACGGTACCATGGACATCATGAGTGAAGT | KpnI | Insert *xplB* into pSAT4a and pnSAT3a |
| XplbR1 | ttGGATCCTCAGCAGACCGATTCGGCCGGC | BamHI |  |
| NrF1 | TCGAATTCAACAATGGATATCATTTCTGTCG | EcoRI | Insert *nfsI* gene into pSAT7a and pSAT4a |
| NrR1 | TTGGATCCTCAGCACTCGGTCACAATCG | BamHI |  |
| OsActinF1 | TTACCGGTCTCGAGGTCATTCATAT | AgeI | Clone and insert *Os*actin promoter into pSAT1a to produce pnSAT1a |
| OsActinR1 | TTAAGCTTTCTACCTACAAAAAAGCTCC | HindIII |  |
| ZmUbiF1 | TTACCGGTTGCAGTGCAGCGTG | AgeI | Clone and insert *Zm*Ubi promoter into pSAT3a to produce pnSAT3a |
| ZmUbiR1 | CCAAGCTTTGCAGAAGTAACACC | HindIII |  |
| PvUbiF1 | TTACCGGTCCACTGGAGAGGG | AgeI | Clone and insert *Pv*Ubi promoter into pSAT6a to produce pnSAT6a |
| PvUbiR1 | TTAAGCTTGATCTGCATCTGCAGAAG | HindIII |  |
| GFPF5 | TTAAGCTTATTATGGTAGATCTGACTAGT | HindIII | Clone and insert GFP gene into pnSAT1a |
| GFPR5 | ATTCTGCAGTCACACGTGGTGGTGG | PstI |  |
| GUSF4 | TTGGAATTCATTATGGTAGATCTG | EcoRI | Clone and insert GUS gene into pnSAT3a and pnSAT6a |
| GUSR4 | TTAGGATCCTCACACGTGGTG | BamHI |  |
| Sat4seqf1 | CGAATCTCAAGCAATCAAGC |  | PCR to confirm the insertion of *hpt* cassette of prcs2-abnr-hr vector in grass genome DNA. |
| Sat4seqr1 | CCTTATCTGGGAACTACTCAC |  |  |
| nSAT1aseqf1 | GCTGCTTCGTCAGGCTTAGAT |  | Work Together with SAT4aseqr1 to do PCR to confirm the insertion of *hpt* cassette of prcs2-NABNR vector in grass genome DNA. |
| NSAT3aseqf1 | CTTGATATACTTGGATGATGGC |  | PCR to confirm the insertion of *xplB* cassette of prcs2-NABNR vector in grass genome DNA. |
| Sat3aseqr1 | AGCCACGCACATTTAGGA |  |  |
| NSAT6aseqf1 | TGCTGTGATGCTGTTTGTTG |  | PCR to confirm the insertion of *xplA* cassette of prcs2-NABNR vector in grass genome DNA. |
| Sat6aseqr1 | CCGGAAACAAACAACGA |  |  |
| Xpla300f | CAACAACGCGATCGACATCC |  | qRT-PCR of *xplA* gene |
| Xpla959r | TCGAACATCGCCTCCATCAC |  |  |
| XplB511F | GAATTCACCGGTTCCGATCT |  | qRT-PCRR of *xplB* gene |
| XplB1143R | GACTGTCCGGTCGATCACTT |  |  |
| Nfs1322f | TTCAACACGCCAGAAGCCA |  | qRT-PCR of *nfsI* gene |
| Nfs1652r | AGCACTCGGTCACAATCGT |  |  |
| HptF2 | TCTTAGCCAGACGAGCGGGTTC |  | qRT-PCR of *hpt* gene |
| HptR2 | TACTTCTACACAGCCATCGGTCCAG |  |  |
| eIF-4aF | TGATGTCATTCAGCAAGCACAA |  | eukaryotic initiation factor-4A (*eIFa*) |
| eIF-4aR | GGCATTCAACCAGGCCATAG |  |  |

**Supplemental methods**

**Biolistic bombardment of epidermal onion cells**

Biolistic bombardment was carried out using a PDS1000He biolistic gun (Bio-Rad). The onion was positioned at 10 cm target distance. The micro projectiles were bombarded at a rupture pressure of 1100 psi. The bombarded onion was wrapped with wet paper towels and kept in a culture vessel at room temperature in the dark for two days before observation.

**GUS assay**

The epidermal layer of onion cells was peeled off and assayed for GUS activity. The GUS incubation buffer consisted of 2 mm x-gluc (5-bromo-4-chloro-3-indolyl-β-D-glucuronic acid), 2 mm potassium ferricyanide and potassium ferrocyanide, 50 mm sodium phosphate buffer ph 7.2, 0.2% Triton X-100. The tissues were soaked in GUS incubation buffer at 37 °C overnight and then kept in 70 % ethanol and observed by light microscopy.

**GFP observations**

Two days after bombardment the epidermal cells of onion were visualized using a laser scanning confocal microscope (LSM5 PASCAL, Zeiss). The detection limits of the microscope were set using images captured from tissue that was not bombarded with GFP. The excitation wavelength was 448 nm, and the images collected through TRITC filters for GFP fluorescence.
